# Effort–Reward Imbalance Among Emergency Department Nurses in China: Construction and Evaluation of a Nomogram Predictive Model

**DOI:** 10.1155/jonm/1412700

**Published:** 2025-06-05

**Authors:** Luying Zhong, Ling Wang, Hao Zhang, Dongmei Diao, Xiaoli Chen, Liqun Zou

**Affiliations:** ^1^Department of Emergency Medicine, West China Hospital, Sichuan University/West China School of Nursing, Chengdu, China; ^2^Disaster Medical Center, Sichuan University, Chengdu, China; ^3^Nursing Key Laboratory of Sichuan Province, Chengdu, China

**Keywords:** cross-sectional study, effort–reward imbalance, emergency department nurses, predictive model

## Abstract

**Background:** Emergency department nurses face severe occupational stress. Effort–reward imbalance (ERI) has been shown to be a significant psychosocial stressor closely linked to adverse health consequences.

**Objectives:** The primary objective of this study was to construct and rigorously evaluate a predictive model for ERI in emergency department nurses. The model is intended to precisely identify high-risk populations and provide a crucial reference in the formulation of targeted intervention strategies.

**Design:** A descriptive cross-sectional survey design was employed.

**Methods:** The study sample comprised 1540 registered nurses from 30 tertiary hospitals in China. The demographic characteristics of the respondents, their responses to the Chinese version of the ERI questionnaire, and their responses to the Chinese Nursing Work Environment (C-NWE) scale were collected via an anonymous online questionnaire. We used multiple logistic regression to develop our predictive model. Subsequently, a nomogram was plotted to simplify the model, and its performance was comprehensively evaluated using the area under the curve (AUC) and bootstrap resampling.

**Results:** The prevalence of ERI among emergency department nurses was determined to be 26.2%. Overcommitment and weekly work hours (≥ 59 h) were identified as independent predictors of ERI. The AUC of the model reached 0.891, demonstrating robust discriminatory power.

**Conclusions:** We constructed a precise predictive model that accurately quantifies the contributions of overcommitment and weekly work hours (≥ 59 h) to the risk of ERI among emergency department nurses. These findings have significant implications for the early identification and effective prevention of ERI in high-stress nursing environments.

**Implications for Nursing Management:** Healthcare administrators can use our model to identify nurses at high risk of ERI. By taking steps to address overcommitment and manage work hours, they can mitigate the negative impact of ERI, thereby improving the health of emergency department nurses and enhancing the quality of care.

## 1. Introduction

The emergency department serves as the front line of a hospital, where acute, critical, and severe patients are treated. Emergency department nurses are the mainstay of the emergency medical service and play a crucial role in ensuring the efficient operation of the department. The challenges they face include long working hours, heavy workloads, high work intensity, and a high-pressure working environment. The onerous job responsibilities and harsh working environment expose emergency department nurses to long-term distress, and they need to obtain emotional and financial support from their work to counteract the impact of the working environment. When the rewards received are not sufficient to compensate for the effort invested, it results in effort–reward imbalance (ERI) [1]. ERI is a theoretical model of psychosocial work environments that adversely affect health and well-being. According to the ERI model, the effort at work is primarily the observable workload and the increase in workload in the near future, including time, mental and physical effort, while the reward at work includes money, respect, recognition, and career opportunities. A lack of reciprocity between giving and receiving at work (e.g., high giving and low receiving) can lead to negative emotions, which in turn can lead to persistent changes in the neuroendocrine system and ultimately affect health. ERI in emergency department nurses has been correlated with chronic depression, physical and mental exhaustion, and a reduced sense of accomplishment [2, 3], ultimately leading to the intention to leave the job [4]. This, in turn, results in a decline in the quality of medical services and emergency healthcare staffing shortages. Therefore, addressing ERI among emergency nurses is of utmost importance to the quality of emergency nursing care and the well-being and career stability of the emergency nursing staff.

The occupational stress caused by ERI affects the health of the working population. Research has found that ERI is associated with increased risks of coronary heart disease, depression, diabetes, and substance abuse [5–8]. A study in China found that 59.6% of emergency department nurses suffer from ERI [9]. Factors associated with ERI in emergency department nurses include sex, age, educational level, marital status, hospital type, and night-shift frequency [10]. Given the high prevalence and significant impact of ERI among this population, early identification of those affected is of critical importance. However, to date, there have been few studies on ERI among emergency department nurses. Those that exist have primarily investigated the epidemiology of ERI rather than attempting to gain a deeper understanding of the issue. There is also a dearth of efficient and accurate clinical prediction models to identify emergency department nurses at high risk of ERI.

Therefore, this study will screen factors associated with ERI among emergency department nurses, gleaned from a literature review and clinical experience. Once those variables correlated with a high risk of ERI have been identified, they will be used to develop a prediction model. The study aims to create a means of early identification of nurses with a high risk of ERI. It is hoped that this will facilitate the provision of more effective support and the formulation of intervention management policies. It is expected to provide timely support to emergency nurses, protect their physical and mental health, and prevent potential detrimental effects on the quality of emergency nursing care.

## 2. Materials and Methods

### 2.1. Study Design and Participants

A cross-sectional study was conducted in China between December 26, 2023, and January 18, 2024. Based on the geographical distribution of the Chinese population, 30 tertiary hospitals were selected using stratified cluster random sampling. These comprised three hospitals from the Northeast, four from the North, six from the East, four from the Central region, five from the South, four from the Northwest, and four from the Southwest. All of the emergency department registered nurses included in our sample were aged ≥ 18 years, had ≥ 1 year of experience working in an emergency department, had no documented history of mental illness, and had not used any kind of psychotropic medication in the week preceding the investigation. According to the cross-sectional (qualitative) sample size calculation formula, the ERI detection rate of the pre-survey nurses' group was 26.5%–93% [10, 11], and under the condition of significance level *p* < 0.05 and permissible error of 0.05, the required sample size calculated based on the preliminary pilot survey was 100–300, and the maximum sample size of 300 was selected, which was enlarged by 10% in order to avoid the impact caused by the loss of the sample size, and the final sample size was 330. After the exclusion of 11 incomplete questionnaires (0.71%), a total of 1540 eligible questionnaires were included in our analysis. Consequently, the valid response rate was 99.29%.

### 2.2. Ethics and Informed Consent

This study was conducted in accordance with the tenets of the 1964 Declaration of Helsinki and its later revisions. It was approved by the Institution Review Board of West China Hospital of Sichuan University [approval no. 2024 (309)]. All participating nurses signed an informed consent form before the study began. They were told that they could withdraw at any time and assured of the confidentiality of their information.

### 2.3. Data Collection

To recruit participants, an electronic invitation and a copy of the questionnaire were sent to the Nursing Department Director or the Head Nurse of the Emergency Department at each of the 30 tertiary hospitals selected for inclusion. We were assisted in obtaining the consent of these institutions by the Chinese Nursing Association. The invitation letter requested the hospital's consent to their nurses' participation, with an assurance that the study would be conducted in accordance with high ethical standards. Upon institutional approval, an electronic questionnaire was distributed to the nurses of each emergency department via a network platform (https://www.wjx.cn) with the assistance of the nursing administrators. All questionnaires were completed anonymously by the participants. Before the survey, the nurses were provided with a comprehensive explanation of the purpose of the study, its significance, the concept of ERI, and the confidentiality of their information. An informed consent form was provided with this information. Those nurses who chose to participate completed the survey via WeChat/QQ by clicking on a link or scanning a QR code. A restriction was imposed during the creation of the survey so that each IP address could only submit the survey responses once. Prior to the distribution of the questionnaires, the principal investigator trained the investigators. During the questionnaire collection process, the investigators were responsible for following up on any incomplete or missing responses to ensure fully completed questionnaires. After the questionnaires were collected, the principal researcher was responsible for the collation and quality control of the questionnaires.

### 2.4. ERI, Variables, and Instruments

#### 2.4.1. The ERI Scale

The ERI questionnaire used in this study, which was Chinese localization administered by Jian Li, is considered a reliable and valid instrument for measuring the psychosocial work environment of Chinese workplace professionals [12]. The ERI model used in the questionnaire includes three dimensions: effort (items 1–6), reward (items 7–17), and overcommitment (items 18–22). Each item is scored on a five-point Likert scale, ranging from 1 (completely disagree) to 5 (completely agree). The 17 items are equally weighted, and the presence and degree of job strain are determined by the ERI ratio, calculated as ERI = (effort score/reward score) × (11/6), where 11/6 is a correction factor used to adjust for the discrepancy in the number of effort and reward items. Values close to 0 indicate relatively low effort and relatively high rewards, while values greater than 1.0 indicate a significant amount of effort with insufficient reward. The higher the ratio, the greater the discrepancy between high effort and low reward. In this study, the Cronbach's α coefficients for the ERI and its three subscales were all greater than 0.8, with values of 0.957, 0.894, 0.952, and 0.922, respectively.

#### 2.4.2. Demographic and Occupational Characteristics of Participants

The participant demographic data gathered by our survey comprise information on gender, age, marital status, childbearing status, highest level of education attained, smoking habits, and drinking habits. Occupational data were the type of hospital in which they work, their professional title, years of service (based on Jiang Yan's three-track five-ladder nursing career ladder system), weekly working hours (in accordance with Chinese labor laws, which stipulate a 5-day work week, with 8 h of work per day), night shift circumstances (presence and frequency of night shifts), and monthly income.

#### 2.4.3. The Chinese Nursing Work Environment (C-NWE) Scale

The C-NWE scale was developed in 2016 by Shao et al. [13]. Based on the characteristics of nursing work environments in China, it consists of seven dimensions: professional development, leadership and management, physician-nurse relationships, recognition, professional autonomy, basic guarantees, and staffing, with a total of 26 items. Each item is scored on a six-point Likert scale, ranging from 1 (strongly disagree) to 6 (strongly agree). The possible scores range from 26 to 156, with higher scores indicating greater satisfaction with the work environment. On the Likert scale, a rating of 3 means “somewhat agree.” When this is multiplied by the 26 items, it gives a score of 78. We took this as the cut-off value for satisfaction with the work environment. Hence, those with scores of 78 or above were classed as satisfied and those with scores below 78 were classed as dissatisfied. In this study, the Cronbach's coefficient for the C-NWE scale was 0.981, and the Cronbach's α coefficients for the seven dimensions ranged from 0.927 to 0.964.

### 2.5. Statistical Analysis

Categorical variables were described as composition ratios (%), and continuous variables as the arithmetic mean (M) and standard deviation. Univariate analyses were performed using the chi-square test and the Mann–Whitney U test to screen for potential influences on ERI among emergency department nurses. Multivariate binary logistic regression analysis was then performed to clarify the related variables and construct the ERI prediction model. Finally, the variables identified as correlates of ERI were presented as a nomogram of the prediction model. Odds ratios (ORs) and 95% confidence intervals (CIs) were used to quantify the effect size estimates. In this analysis, the dependent variable was the presence or absence of ERI among emergency department nurses, and the independent variables included demographic characteristics, work characteristics, and scores on the C-NWE scale. To assess the stability and reliability of the model, we employed bootstrap resampling with 1000 bootstrap resamples. To evaluate the model fit, we utilized a combination of the Hosmer–Lemeshow test and the *R*^2^ statistic. All statistical descriptions and analyses were conducted using SPSS for Windows, v.26.0 (IBM Corp., Armonk, NY, USA), and R software (v.4.3.3, https://www.r-project.org/). Two-sided statistical tests were employed. The significance level was set at *p* < 0.05.

## 3. Results

We surveyed 1540 emergency department nurses. The majority were employed in tertiary comprehensive hospitals. They were predominantly female, aged between 20 and 39 years old, and most were married and with children. The majority held a bachelor's degree or higher, with professional titles mainly at the junior level. Most had ≤ 10 years of experience in emergency work. Full details of participants' demographic and employment characteristics are provided in Table 1.

The rate of ERI among the emergency department nurses surveyed was 26.2%.  Table 2 presents the effort-reward ratios (ERR) identified among the participants.

Univariate analysis revealed several variables associated with ERI among the emergency department nurses surveyed. These were the type of hospital in which the nurse worked, age, marital status, highest education level, professional title, work experience, weekly working hours, night shift frequency, and satisfaction with the work environment (Table 3).

Multifactorial logistic regression analysis found overcommitment (OR = 1.635, 95% CI: 1.545–1.731) and weekly work hours ≥ 59 h (OR = 2.594, 95% CI: 1.156–5.818) to be independent predictors of ERI among emergency department nurses. For each one-point increase in the intrinsic engagement score, the risk of ERI increased by 63.5%. The risk of ERI for emergency department nurses working ≥ 59 h per week was 2.594 times higher than for those working ≤ 40 h per week. Detailed results of the multifactorial regression analysis are shown in Table 4.

**Figure 1:**
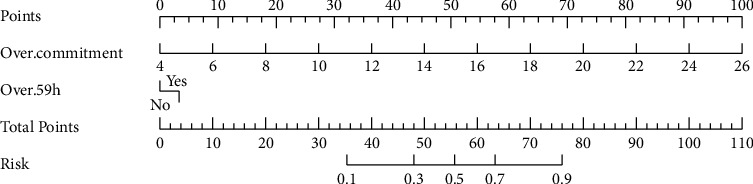
Nomogram of our prediction model for ERI among emergency department nurses.

Using the independent predictive factors for ERI identified in the multifactorial logistic regression (overcommitment and working ≥ 59 h per week), a predictive nomogram model was constructed using R software (Figure 1).

**Figure 2:**
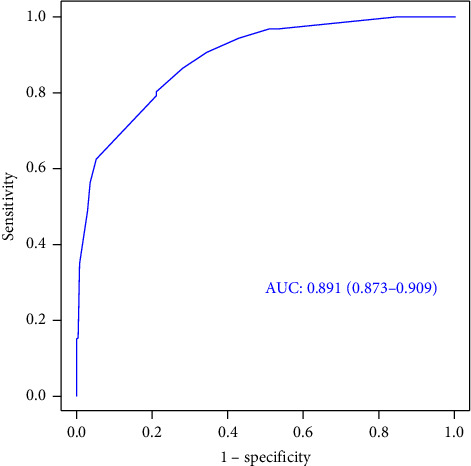
ROC curves for our prediction model for ERI among emergency department nurses.

**Figure 3:**
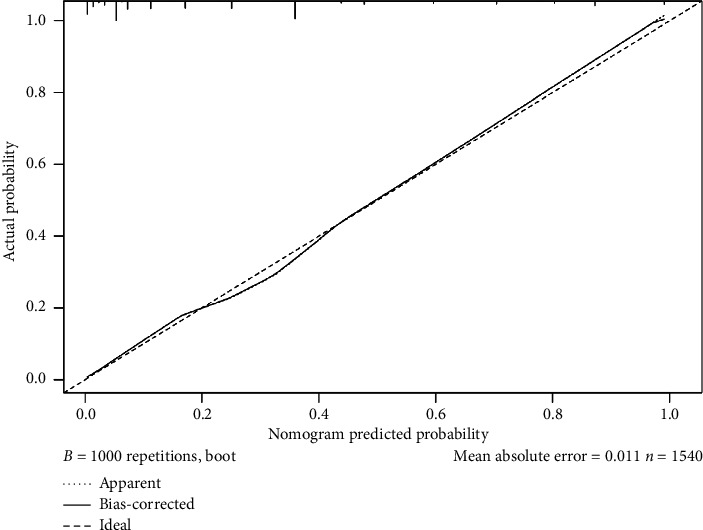
Calibration curves for the nomogram of our prediction model for ERI among emergency department nurses.

A receiver operating characteristic (ROC) curve of the model was plotted, and the area under the curve (AUC) was 0.891 (95% CI: 0.873–0.909) (Figure 2). Using the bootstrap method of resampling with replacement of 1000 times, a calibration curve was plotted. As shown in Figure 3, the calibration curve of the nomogram model is close to the ideal model.

**Figure 4:**
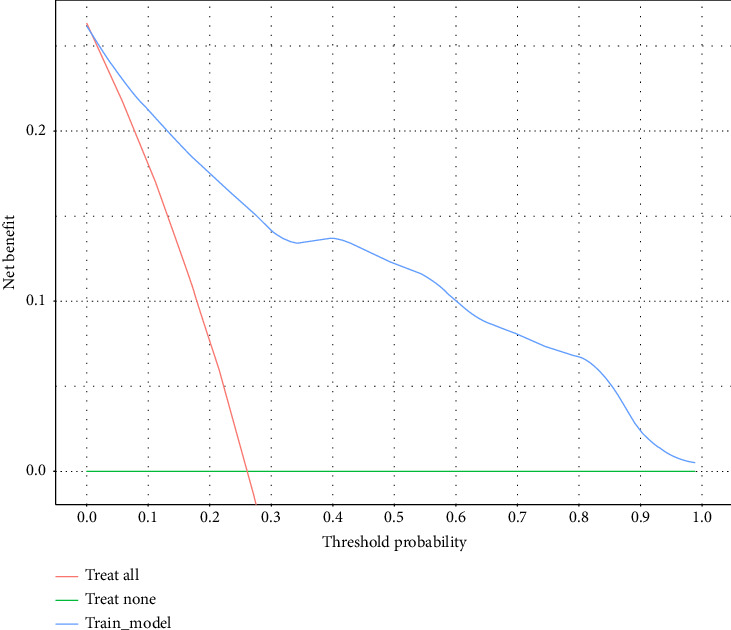
Decision curve analysis of the net value of our prediction model for ERI among emergency department nurses in clinical practice.

To determine the clinical value of our nomogram model, a decision curve was created to evaluate the clinical net benefit of using the nomogram model to predict the risk of ERI among emergency department nurses. The results are shown in Figure 4. With a threshold probability range of 0.01–1, the predictive model curves lie above both the treat—all line and treat—none line, the use of the model was shown to offer net benefits in clinical practice, and a reduction in missed diagnoses.

To assess the prediction model, we evaluated our regression analysis using the Hosmer–Lemeshow test. The results confirmed that the regression model had adequate fit (*χ*^2^ = 11.317, *p*=0.125), indicating no significant deviation from expected values. The internal validity of the predictive nomogram's precision was confirmed using bootstrap resampling, with 1000 resampled datasets.

## 4. Discussion

Although ERI can affect those in any profession, its rate in nurses is particularly high ERI [14, 15]. Emergency department nurses are a critical component of the healthcare workforce. However, they are highly vulnerable to traumatic events, patient-provider conflicts, and mass casualty incidents. These challenges increase the risk of ERI among emergency nurses. We found an ERI prevalence among our sample of 26.2%. This is close to the 26.5% rate found among 1,077 nurses from the Shandong province, but lower than the 59.66% rate reported in another study [5, 11]. Surveys of 257 nurses in Egypt and 389 in Germany found that 72.5% and 77.2%, respectively, were experiencing ERI [16, 17]. A study of emergency department nurses in the United States found an even higher ERI rate of around 93% [10]. These discrepancies between studies can be attributed to variations in factors such as regional economic conditions, healthcare resources, hospital corporate cultures, emergency care workloads, and individual differences between nurses.

Emergency departments are a unique work environment characterized by high stress, intensity, pace, and volume, all of which may potentially contribute to ERI. Therefore, hospital administrators must use risk markers to promptly identify those emergency department nurses at high risk for ERI and provide appropriate preventive strategies or interventions to this vulnerable group to avoid, reduce, or eliminate ERI. At the minimum, it must not be allowed to escalate or persist sufficiently to an extent that may harm nurses' psychological well-being and reduce the quality of their work.

We found weekly working hours to be associated with a risk of ERI. This risk was 2.594 times higher among the nurses with weekly working hours ≥ 59 h than those with weekly working hours ≤ 40 h (95% CI: 1.156–5.818). Working hours have previously been shown to have significant negative effects on the physical and mental health of employees [18, 19]. Longer working hours prevent workers from fully recovering from physical and mental fatigue between work days, leading to increased levels of such fatigue and depletion of emotional resources. This is detrimental to the worker's health, subjective well-being, and job satisfaction [20]. Long working hours are strongly correlated with adverse outcomes among nurses and can even lead to adverse medical events [21]. This may cause further damage to the nurse, including financial and emotional losses and ERI. There are multiple potential mechanisms by which long working hours increase the risk of ERI. One study found that respondents in high-stress jobs with high ERI reported lower work time management compared to respondents in other work situations [22]. Lacking feelings of self-competence can lead to reduced work time management, prolonging work hours. These longer hours are then perceived as evidence of self-competence deficiency, which creates a negative feedback loop. Nurses invest more effort and commit to more hours to improve their competence and time management, leading to ERI.

Emergency nursing managers can utilize our findings proactively by ensuring appropriate work intensity, staff matching of emergency department nurses, and delivering nurse competence training to circumvent the occurrence of ERI among emergency nurses.

Overcommitment describes a work state characterized by excessive effort and a strong desire for recognition [23]. We observed a significant positive correlation between overcommitment and the risk of ERI. Thus, the risk of ERI increases as workers' commitment to their work increases. This is consistent with the results of an 8-year study of skilled professionals in Finland, which found overcommitment to be associated with low returns and an increased risk of ERI [24]. A large cohort study of 3782 men and 3731 women from the Czech Republic, Poland, and Russia revealed a complex relationship between overcommitment and ERI. While the relationship was bidirectional, the impact of overcommitment on ERI was significantly stronger than the impact of ERI on overcommitment [25]. However, a population-based cohort study from Switzerland found no significant correlation between overcommitment and ERI [26]. This discrepancy may stem from economic and cultural differences between countries. In work requiring a great deal of physical labor, the relationship between overcommitment and ERI is particularly pronounced. Another previous study has shown that workers in the manufacturing and service sectors often experience low rewards for considerable effort. Again, this was significantly associated with overcommitment [27]. When workers who are excessively devoted to their jobs do not receive commensurate returns, it can lead to the onset or exacerbation of ERI. Conversely, persistent ERI may push an individual to overcommit in an attempt to compensate for the imbalance by working even harder.

Liang et al. have shown that overcommitment in nurses has a significant direct effect on ERI and an indirect effect on the quality of working life, leading to decreased job satisfaction and quality of life [28]. Like ERI, excessive commitment is particularly pronounced among emergency department nurses, who often go to great lengths for their patients, pushing beyond their physical and psychological limits to provide the best possible care and save lives [10]. However, this high degree of commitment can have a negative impact on physical and mental health [29], increasing their risk of ERI. Nursing managers should pay close attention to signs of overcommitment among emergency department nurses and take measures to improve the work environment and working conditions. Among other strategies, reducing work pressure, optimizing workflow, providing timely psychological assistance, and offering professional guidance can help to reduce overcommitment among nurses. This can enhance their job satisfaction and quality of life. Notably, overcommitment and ERI are also associated with menstrual pain in women [30], which is particularly important in nursing populations, which are predominantly composed of young women. Furthermore, high ERI and overcommitment scores increase the risks of stress-related illnesses later in life and the occurrence of medical errors [7, 31, 32]. Emergency department managers and occupational health services should be fully aware of these associations and take proactive steps to reduce work stress and maintain the physical and mental health of nurses to reduce the incidence of medical errors.

The visual aid we have developed, in the form of a nomogram, offers a straightforward, customized approach to ERI forecasting that can enhance the efficiency of nursing management. The reliability of our predictive framework was substantiated by an internal validation process utilizing bootstrap resampling. Weekly work durations exceeding 59 h and overcommitment were identified as risk factors for ERI. These were integrated into our prediction model. It is important to acknowledge that the generalizability of our model has not been established, as it has yet to undergo external validation.

The model serves as a valuable instrument for the identification of emergency department nurses at heightened risk of ERI through the targeted evaluation of risk factors. In practice, the column-line graphs can be synergized with established assessment tools to furnish a thorough evaluation of each nurse's condition. For instance, a nurse working fewer than 59 h per week with no signs of overcommitment would register as a null risk on our graphical representation. However, this does not eliminate the possibility of future ERI development. Through estimation of the likelihood of ERI occurrence and identification of particular risk factors, our model can assist in the formulation of strategic countermeasures against ERI within the emergency nursing community.

Our study has several limitations. First, our predictive model has not yet been subjected to external validation. Nonetheless, our internal evaluation using resampling methods and calibration of the predicted probability of ERI to a certain extent compensates for this shortcoming. Second, our study relied primarily on scale measurements, so the reliability and validity of each scale used should, ideally, be assessed using multiple approaches, but we only reported Cronbach's α. Third, the generalizability of our model to emergency department nurses outside of China is uncertain because of variations in nursing practices and healthcare systems between countries. Furthermore, the classification of some variables within our study was contingent on the researchers' discretion. Different categorization approaches could yield different outcomes. Lastly, the distribution of questionnaires through directors of nursing and head nurses may have introduced bias into our study population. This could have led to partial nondisclosure by participants, particularly concerning sensitive matters such as effort and reward.

## 5. Conclusions

We built a parsimonious model based on emergency department nurses' weekly work hours and level of overcommitment to predict their risk of developing ERI. The model was well-calibrated. The resulting nomogram can be used to screen emergency department nurses for their risk of developing ERI and assist organizations, administrators, and care managers in the implementation of more effective, targeted ERI prevention strategies within the emergency department nursing workforce.

## 6. Implications for Nursing Management

Results of the study indicate that excessive weekly hours and overcommitment are independent factors in the ERI nomogram predictive model for emergency department nurses. Healthcare administrators can utilize our model to accurately identify nurses at high risk for ERI in the emergency department. For these high-risk groups, healthcare organizations mitigate their overwork problems by implementing targeted strategies, including physical and psychological overwork, which should be scientifically planned for work hour management, timely communication, and psychological counseling, in order to reduce the negative impacts of ERI, improve the physical and mental health of emergency department nurses, safeguard the human resources in emergency medicine, and further optimize the quality of nursing services, thereby providing patients with safer and more efficient emergency medical services.

## Figures and Tables

**Table 1:** Demographic and employment information of the study participants (*n* = 1540).

Variable	Category	*n*	%
Nature of the hospital of employment	Tertiary specialized hospital	23	1.5
	Tertiary general hospital	1517	98.5

Age stratification	20–29 years old	582	37.8
	30–39 years old	734	47.7
	40–49 years old	183	11.9
	≥ 50 years old	41	2.7

Gender	Female	1211	78.6
	Male	329	21.4

Marital status	Single	560	36.4
	Married	980	63.6

Fertility status	Childless	710	46.1
	Fertile	830	53.9

Highest level of education	Junior college	186	12.1
	Bachelor's degree or above	1354	87.9

Professional title	Junior	907	58.9
	Intermediate	574	37.3
	Senior	59	3.8

Work experience stratification	≤ 5 years	491	31.9
	6–10 years	493	32.0
	11–15 years	300	19.5
	16–20 years	121	7.8
	≥ 21 years	135	8.8

Weekly work hours	≤ 40 h	577	37.5
	41–48 h	780	50.6
	49–58 h	123	8.0
	≥ 59 h	60	3.9

Night shift	Yes	1359	88.2
	No	181	11.8

Night shift frequency	0 times/month	181	11.8
	1–4 times/month	239	15.5
	5–8 times/month	659	42.8
	> 8 times/month	461	29.9

Monthly income	< ¥14,000/month	57	3.7
	¥4000–5999/month	149	9.7
	¥6000–7999/month	250	16.2
	¥8000–9999/month	368	23.9
	≥¥10,000/month	716	46.5

Smoking status	Nonsmoker	1453	94.4
	Smoker	87	5.6

Drinking status	Nondrinker	1355	88.0
	Drinker	185	12.0

Satisfaction with the nursing work environment	Unsatisfied	73	4.7
	Satisfied	1467	95.3

*Note:* ¥, Chinese currency symbol.

**Table 2:** Effort–reward ratios for emergency department nurses (*n* = 1540).

Variable	Category	*n*	%
Effort–reward ratio	> 1 (imbalance)	403	26.2
	= 1 (balance)	105	6.8
	< 1 (low effort-high reward)	1032	67.0

**Table 3:** Results of univariate analyses of variables that may affect effort–reward imbalance among emergency department nurses.

Variable	Category	ERR > 1	ERR ≤ 1	*χ* ^2^/*Z*	*p*
		*n* = 403	*n* = 1137		
Nature of the hospital of employment	Tertiary specialized hospital	0	23	8.276	0.003
	Tertiary general hospital	403	1114		

Age stratification	20–29 years old	126	456	13.661	0.003
	30–39 years old	208	526		
	40–49 years old	61	122		
	≥ 50 years old	8	33		

Gender	Female	323	888	0.743	0.397
	Male	80	249		

Marital status	Single	124	436	7.382	0.007
	Married	279	701		

Fertility status	Childless	172	538	2.575	0.116
	Fertile	231	599		

Highest level of education	Junior college	30	156	11.037	0.001
	Bachelor's degree or above	373	981		

Professional title	Junior	209	698	19.401	0.000
	Intermediate	166	408		
	Senior	28	31		

Work experience stratification	≤ 5 years	103	388	14.210	0.007
	6–10 years	133	360		
	11–15 years	83	217		
	16–20 years	43	78		
	≥ 21 years	41	94		

Weekly work hours	≤ 40 h	107	470	47.75	0.000
	41–48 h	215	565		
	49–58 h	53	70		
	≥ 59 h	28	32		

Night shift	Yes	354	1005	0.087	0.787
	No	49	132		

Night shift frequency	0 times/month	49	132	10.956	0.012
	1–4 times/month	48	191		
	5–8 times/month	163	496		
	> 8 times/month	143	318		

Monthly income	< ¥4000/month	20	37	3.929	0.415
	¥4000–5999/month	42	107		
	¥6000–7999/month	63	187		
	¥8000–9999/month	101	267		
	≥ ¥10,000/month	177	539		

Smoking habits	Nonsmoker	383	1070	0.483	0.532
	Smoker	20	67		

Drinking habits	Nondrinker	352	1003	0.213	0.656
	Drinker	51	134		

Satisfaction with work nursing environment	Unsatisfied	32	41	12.936	0.000
	Satisfied	371	1096		

Overcommitment		17 (15,20)	11 (9,14)	−23.457	0.000

*Note:* ¥, Chinese currency symbol; *χ*^2^, chi-square value; Z, standardized normal statistic; *p*, probability.

Abbreviation: ERR, effort–reward ratio.

**Table 4:** Results of binary logistic regression analysis of variables that may affect effort–reward imbalance among emergency department nurses.

	*B*	SE	Waldc^2^	*p*	OR	95% CI	
Constant	−27.965	6830.240	0	0.997	0		

*Nature of the hospital of employment*							
(Ref: Tertiary specialized hospital)					1		
Tertiary general hospital	19.227	6830.235	0.000	0.998	223,870,547.185	0.000	—

*Age stratification*							
(Ref: ≤ 20 years old)					1		
30–39 years old	0.087	0.328	0.070	0.792	1.090	0.573	2.074
40–49 years old	0.028	0.550	0.003	0.960	1.028	0.350	3.024
≥ 50 years old	−0.967	0.813	1.416	0.234	0.380	0.077	1.870

*Marital status*							
(Ref: Single)					1		
Married	0.246	0.226	1.184	0.277	1.279	0.821	1.990

*Highest level of education*							
(Ref: Junior college)					1		
Bachelor's degree or above	0.332	0.287	1.333	0.248	1.393	0.793	2.447

*Professional title*							
(Ref: Junior)					1		
Intermediate	−0.151	0.203	0.549	0.459	0.860	0.578	1.281
Senior	−0.093	0.434	0.046	0.830	0.911	0.389	2.135

*Work experience stratification*							
(Ref: ≤ 5 years)					1		
6–10 years	0.075	0.332	0.051	0.821	1.078	0.562	2.068
11–15 years	−0.057	0.406	0.02	0.888	0.944	0.426	2.094
16–20 years	0.303	0.518	0.342	0.558	1.354	0.491	3.734
≥ 21 years	0.548	0.647	0.716	0.397	1.729	0.486	6.145

*Weekly work hours*							
(Ref: ≤ 40 h)					1		
41–48 h	0.246	0.178	1.906	0.167	1.279	0.902	1.812
49–58 h	0.512	0.289	3.128	0.077	1.668	0.946	2.941
≥ 59 h	0.953	0.412	5.349	**0.021**	2.594	1.156	5.818

*Night shift frequency*							
(Ref: 0 times/month)					1		
1–4 times/month	0.083	0.323	0.065	0.798	1.086	0.576	2.047
5–8 times/month	−0.012	0.286	0.002	0.965	0.988	0.564	1.730
> 8 times/month	0.102	0.292	0.123	0.726	1.108	0.625	1.965

*Satisfaction with work environment*							
(Ref: Satisfied)					1		
Unsatisfied	0.093	0.428	0.047	0.828	1.098	0.475	2.539
Overcommitment	0.492	0.029	287.731	**0.000**	1.635	1.545	1.731

*Note:* B, regression coefficient; *p*, probability.

Abbreviations: CI, confidence interval; OR, odds ratio; SE, standard error.

## Data Availability

The data that support the findings of this study are available from the corresponding author upon reasonable request.
